# RESIDUAL LIMB NEUROPATHIC PAIN ASSOCIATION WITH NEUROMA, PROSTHETIC, FUNCTION, AND PARTICIPATION OUTCOMES IN INDIVIDUALS LIVING WITH A TRANSTIBIAL AMPUTATION: AN EXPLORATORY STUDY

**DOI:** 10.2340/jrm.v57.40551

**Published:** 2025-01-21

**Authors:** Camille FOURNIER-FARLEY, Mathieu BOUDIER-REVÉRET, Dany H. GAGNON

**Affiliations:** 1 Centre for Interdisciplinary Rehabilitation Research of Greater Montreal (CRIR) – Centre Intégré Universitaire de Santé et de Services Sociaux du Centre-Sud-de-l’Île-de-Montréal, Montréal; 2 Faculty of Medicine, Physical Medicine and Rehabilitation Program, Université de Montréal, Montréal; 3 Physical Medicine and Rehabilitation Service, Department of Medicine, Centre hospitalier de l’Université de Montréal, Montréal; 4 School of Rehabilitation, Faculty of Medicine, Université de Montréal, Montréal, Québec, Canada

**Keywords:** amputation, neuroma, pain, physical functional performance, rehabilitation, ultrasonography

## Abstract

**Objective:**

To determine the strength of the association between residual limb neuropathic pain intensity and the number of neuromas, prosthetic, functional, and participation outcomes, and assess whether ultrasound (US) biomarkers of neuromas differ between pain intensities.

**Design:**

Cross-sectional study.

**Subjects:**

Twenty-two participants with a transtibial amputation for more than 12 months, with and without residual limb neuropathic pain.

**Methods:**

Participants completed questionnaires (Numerical Pain Rating Scale, Pain Disability Index (PDI), Prosthetic Profile of the Amputee-Locomotor Capabilities Index), and had their residual limbs examined by US. Whenever a neuroma was diagnosed during US, images of the neuroma(s) were recorded and US biomarkers were computed.

**Results:**

Of the 27 neuromas diagnosed, pain intensity was associated with no use of walking aid, less daily prosthesis wearing time, a higher PDI score, and a neuroma at the common fibular nerve. The cross-sectional area, the thickness ratio, or the thickness of the overlying tissues was not associated with pain intensity.

**Conclusion:**

Though the results enrich currently available evidence on clinical variables potentially associated with the intensity of neuropathic pain in individuals living with a transtibial amputation, and on the limited value of US biomarkers studied to determine the association between neuroma(s) and pain intensity, future studies providing higher quality evidence remain needed.

## LAY ABSTRACT

Many individuals with lower limb amputations complain of neuropathic residual limb pain caused by neuroma(s). Gaining a better understanding of how this pain is associated with other clinical and imaging outcomes will help to advance physical medicine and rehabilitation practices. The main objectives were to determine the strength of the association between residual limb neuropathic pain intensity and the number of neuromas, prosthetic, functional, and participation outcomes, and assess whether some features of neuromas evaluated by ultrasound (US) differ between pain intensities. Though the results showed that pain intensity was associated with no use of walking aid, less daily prosthesis wearing time, and a neuroma at the common fibular nerve, careful interpretation of the results remains recommended given the strength of the evidence. The features of the neuromas examined by US in this study were not associated with pain intensity.

Approximately 60% of individuals with an amputation experience residual limb pain (RLP) characterized by the presence of localized pain in the remaining part of the amputated limb (1). RLP can be caused by numerous aetiologies, almost half of which are linked to the presence of painful neuromas (2, 3). In amputated limbs, neuromas are a benign proliferation of neural tissue that forms a bulbous tangled mass at the end of a transected nerve (1, 4). These masses can cause neuropathic pain in the form of an electrical shock, particularly when stimulated by pressure (5, 6).

Between 2006 and 2012, transtibial amputations represented 31% of all levels of lower limb amputations performed in Canada (7). Individuals who sustain a lower limb amputation often experience functional disabilities (1). Specifically, individuals with lower limb amputations will encounter challenges during standing, walking, and performance of related functional activities such as sit–stand transfers, and stair ascent and descent. Importantly, the pain associated with neuromas can be triggered by the pressure associated with prosthesis wear. This discomfort can lead to limiting use of the prothesis, furthering these functional disabilities. Therefore, characterizing neuromas is important in the context of a comprehensive physical medicine and rehabilitation assessment in the incorporation of a pain prevention and management. Recent advancements in high-resolution musculoskeletal ultrasonography make it an emerging technique that allows one to easily and rapidly visualize, in real-time, the superficial nerves and characterize their integrity (8, 9).

The primary objective of the present study was to determine the strength of the association between neuropathic RLP intensity (i.e., none, mild, moderate, severe, and extreme) and prosthetic, functional, and participation outcome measures. It was hypothesized that higher pain levels would have a strong association with less frequent prosthetic use, lower functional capacity scores, and lower reports of participation. The secondary objective assessed the strength of the association between neuropathic RLP intensity (i.e., none-to-mild pain vs moderate-to-extreme pain) and ultrasound biomarkers of neuromas, as well as symptomatology upon neuroma compression with the ultrasound transducer. Higher pain level was expected to be strongly associated with neuromas presenting with a larger cross-section area (CSA), a higher thickening ratio, and less overlying tissues, as well as the presence of symptoms upon neuroma compression.

## METHODS

### Participants

All participants were over the age of 18 years and had sustained a transtibial amputation more than a year prior to entering the study. Participants were included in the study regardless of whether neuropathic RLP was reported, and participants were excluded if they presented with a broken skin barrier on the residual limb. Potential participants contacted the research team after having been informed about the project by their local prosthetist during a routine clinical appointment or by a research associate who phoned them after having obtained a list of potential participants from the medical records department. This list was generated through a systematic search of the electronic medical records database, applying predefined inclusion/exclusion criteria. Ethical approval was obtained from the Centre for Interdisciplinary Research in Rehabilitation of Greater Montreal (CRIR-1464-0220) Research Ethics Committee. Participants signed an informed consent before entering the study. All participant-related information and collected data were de-identified and anonymised.

### Clinical evaluation

*Sociodemographics and prosthetic.* History was taken for every participant, including the date and aetiology of amputation, prosthetic wear habits (number of days/week; number of hours/day), use of technical aids, and self-reported ambulatory level (K-levels) (10).

*RLP.* The Numerical Pain Rating Scale (NPRS), ranging from 0 to 10, was used to document the highest level of neuropathic pain experienced in the residual limb during the last week. Before completing this numerical scale, to ensure that only neuropathic pain in the residual limb was assessed, participants were asked to describe their pain. If the pain was characterized as “burning, painful cold sensation or electric shocks”, these participants were classified as having neuropathic pain and instructed to complete the NPRS based on this type of pain. Depending on their NPRS score, participants were classified into 1 of the following 5 categories: none (0), mild (1 to 3), moderate (4 to 6), severe (7 to 9), or extreme neuropathic pain (10, 11). Participants not experiencing neuropathic pain did not complete the NPRS and were classified as having no neuropathic pain (0).

*Function and participation.* A paper version of the Pain Disability Index (PDI) (12, 13) was conducted to measure the impact that pain has on an individual’s participation in 7 categories of life activities: family/home responsibilities, recreation, social activity, occupation, sexual behaviour, self-care, and life-support activities. The Prosthetic Profile of the Amputee-Locomotor Capabilities Index 5 (LCI-5) (14, 15) was also used, which quantified locomotor skills while using the prosthesis. Both indices were self-administered and then reviewed with 1 of the investigators (CFF) prior to the evaluation.

### Ultrasound imaging evaluation

A single experienced examiner (MBR) conducted all musculoskeletal ultrasound assessments of the neuromas using a Philips HD11XE machine and a 5 cm linear transducer (5-12 MHz) (Philips Medical Systems, Bothell, WA, USA). All nerves and their branches, whenever applicable, were localized and scanned until the nerve disappeared or until a neuroma was found. Neuromas were diagnosed and confirmed via the visualization of a hypoechoic mass in continuity with a peripheral nerve. For each neuroma, images in the longitudinal and transverse planes were recorded. During the evaluation, a sonopalpation Tinel test was also performed by applying pressure on each neuroma with the transducer. A positive Tinel test was recorded if a sensation of acute paraesthesia in the distribution of the nerve was reported. The test was deemed negative if no sensation was present.

All recorded images were analysed with a custom-developed program using MATLAB image processing toolbox TM (The MathWorks Inc., Natick, MA, USA) used in previous projects (16). A single evaluator (CFF), who was present during all ultrasound imaging examinations, visualized all images and manually outlined the neuromas to extract key geometric biomarkers including: CSA of the neuroma, thickness of the neuroma in longitudinal plane (short axis of the neuroma), thickness of the proximal nerve in longitudinal plane, and thickness of the overlying soft tissues in both planes. The thickness ratio was computed using the thickness of the neuroma divided by the thickness of the proximal nerve in the longitudinal plane.

### Statistics

For the primary objective, the Goodman and Kruskal’s measure (γ) was computed to determine the association between pain intensity (none, mild, moderate, severe, and extreme) and sociodemographics, prosthetic, function, and number of neuromas and nerve-affected variables (17). This nonparametric measure (γ) ranges from –1 to +1 and indicates both the strength and direction of the association (18). A value of –1 indicates that all pairs of observations are discordant, whereas a value of +1 indicates that all pairs of observations are concordant. Gau (19) recommends interpreting the association as being weak (0 to 0.19), moderate (0.20 to 0.39), strong (0.40 to 0.59), or very strong (0.60 to 1.00). For the secondary objective, the Wilcoxon–Mann–Whitney test, a rank-based nonparametric test, was used to explore whether quantitative musculoskeletal ultrasound biomarkers differed between participants who reported no or mild pain (NPRS < 4) and participants with moderate-to-extreme pain (NPRS ≥ 4) (20). In addition, the χ^2^ test was used to determine if the sonopalpation Tinel sign (positive vs negative) was associated with the participants who experienced none-to-mild pain (NPRS < 4) or those with moderate-to-extreme pain (NPRS ≥ 4). Outcomes reaching *p* ≤ 0.05 were reported as statistically significant. All statistical analyses were computed with SPSS version 26.0 for Windows (IBM Corp, Armonk, NY, USA).

## RESULTS

### Participants

A total of 22 participants were included in the study. Table I summarizes the sociodemographic and personal characteristics of all participants.

**Table I:** Summary of all sociodemographic characteristics and anthropometric measures

Factor	Units	All participants (*n* = 22)			None-to-mild pain (*n* = 13)			Moderate-to-extreme pain (*n* = 9)		

		Q25	Median	Q75	Q25	Median	Q75	Q25	Median	Q75
Age	years	60	62,5	68	62	64	72	52	60	63
Sex, male/female	*n*	6F:16M			1F:12M			5F:4M		
Time since amputation	years	2	2,5	4,75	2	2	3	3	8	23
Aetiology	*n*	13VD:8T:1N			12VD:1T:0N			1VD:7T:1N		
Weight	kg	70,1	85,6	93,0	63,0	86,2	93,0	72,6	77,1	93,0
Height	m	1,7	1,7	1,8	1,7	1,8	1,8	1,6	1,7	1,8

VD: vascular-diabetes; T: traumatic; N: neoplasia.

### Neuromas

A total of 17 of the 22 participants (77%) were diagnosed by ultrasound with the presence of at least 1 neuroma. A total of 27 neuromas were identified among the 22 transtibial residual limbs evaluated. The number of neuromas in each residual limb ranged between 0 and 3. Overall, 11 residual limbs had a neuroma on the superficial fibular nerve, 6 on the deep fibular nerve, 5 on the tibial nerve, 4 on the common fibular nerve, and 1 on the sural nerve.

### Pain and functional capacity measures

When considering the NPRS results, the NPRS < 4 group had a median score of 0, and the NPRS ≥ 4 group had a median score of 7. Overall, most participants reported wearing their prosthesis every day. Only 1 participant in the NPRS < 4 group indicated that they did not wear a prosthesis. Additionally, no participants within the NPRS ≥ 4 group used a walker, and 7 reported that they did not use any walking aid. Regardless of a high or low NPRS score, participants had high LCI-5 scores. Lastly, PDI scores of the participants ranged between 0 and 22 (out of a possible 70) (Table II).

**Table II:** Prosthetic wear, functional locomotion, and pain measures

Factor	Units	All participants (*n* = 22)			None-to-mild pain (*n* = 13)			Moderate-to-extreme pain (*n* = 9)		

		Q25	Median	Q75	Q25	Median	Q75	Q25	Median	Q75
Pain measures										
NPRS (highest)	/10	0	0	6,75	0	0	0	6	7	10
PDI (/70)	/70	0	0	10,75	0	0	0	10	12	16
Prosthetic wear and functional locomotion measures										
Prosthetic wear	(Y/N)	21Y:1N			13Y:0N			8Y:1N		
Prosthetic wear	days/week	7	7	7	7	7	7	4	7	7
Prosthetic wear	hours/day	8,25	11,75	14,88	9	12	15	5,50	9	14,50
Walking aid	*n*	10N:6Ca:4Cr:2W			3N:5Ca:3Cr:2W			7N:1Ca:1Cr:0W		
K-level	0–4	2	2	3	2	2	2	2	3	3
LCI-5	/56	40	45,5	52,75	39	44	46	44	46	56
Number of neuromas	n	1	1	2	1	1	2	1	1	2

LCI-5: Locomotor Capabilities Index – 5; NPRS: Numerical Pain Rating Scale; PDI: Pain Disability Index; N: none; Ca: cane; Cr: crutches; W: walker.

### Neuropathic pain associations

**Fig. 1:**
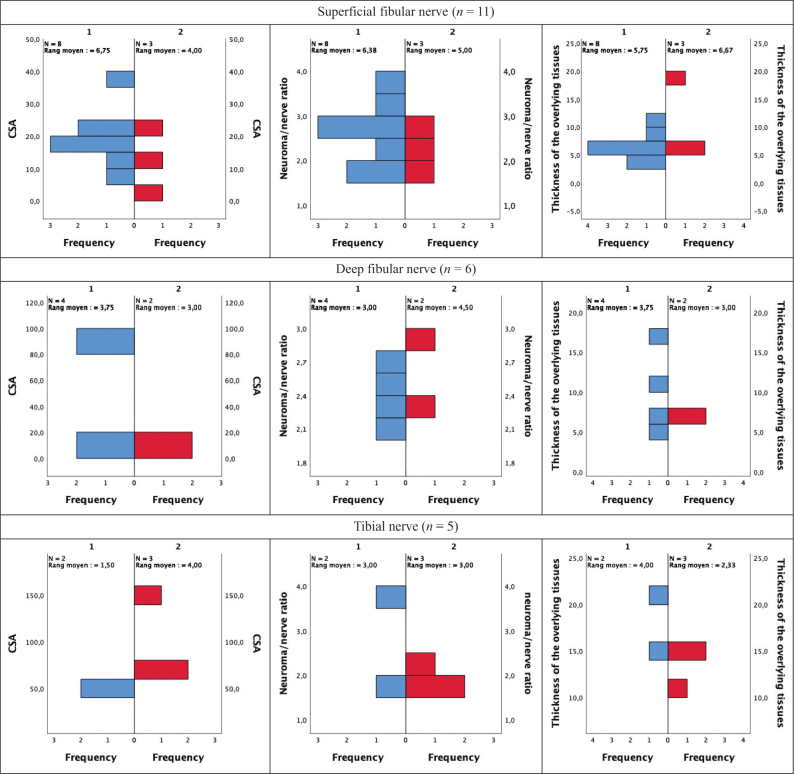
Mann–Whitney *U* tests for independent sample for each biomarker (CSA, neuroma/nerve ratio, and thickness of the overlying tissues) between the 2 groups (Group1/blue = none-to-mild pain; Group 2/red = moderate-to-extreme pain). CSA: cross-sectional area.

Table III summarizes the relationships between neuropathic RLP intensity and characteristics such as prosthetic, functional, and participation measures (i.e., the primary objective). Characteristics such as having a higher PDI score (*p* < 0.000) and the presence of a neuroma on the common fibular nerve (*p* = 0.006) were very strongly, positively, and significantly associated with RLP intensity. Conversely, daily prosthetic wear time (*p* = 0.033) and type of walking aid (*p* = 0.003) were both very strongly and significantly, but negatively associated with the RLP. Indeed, both a prosthesis wear time of 7 days per week and the use of a walker were associated with a lower pain score. Fig. 1 displays results of the secondary objective, which examined the relationship between neuropathic pain intensity and quantitative musculoskeletal ultrasound biomarkers. Overall, the distributions of these biomarkers were comparable between participants who had pain intensities within the none-to-mild range (*n* = 13) and those within the moderate-to-extreme pain scores (*n* = 9), as assessed by visual inspection. In fact, no statistically significant differences were found for the CSA (superficial fibular nerve: *U* = 6, *z* = –1.225, *p* = 0.279; deep fibular nerve: *U* = 3, *z* = –0.463, *p* = 0.800; tibial nerve: *U* = 6, *z* = 1.732, *p* = 0.200), the thickness ratio between the two groups of participants (superficial fibular nerve: *U* = 9, *z* = –0.612, *p* = 0.630; deep fibular nerve: *U* = 6, *z* = 0.926, *p* = 0.533; tibial nerve: *U* = 3, *z* = 0.000, *p* = 1.000), or the thickness of the overlying tissues (superficial fibular nerve: *U* = 14, *z* = 0.408, *p* = 0.776; deep fibular nerve: *U* = 3, *z* = –0.463, *p* = 0.800; tibial nerve: *U* = 1, *z* = –1.155, *p* = 0.400), using an exact sampling distribution for *U* (21). As all participants who had a neuroma identified on the common fibular or the sural nerves expressed moderate-to-extreme pain, group comparison and hypothesis testing were prohibited.

**Table III:** Association between pain intensity and sociodemographics, amputation, and prosthetic, functional ability, and number of neuromas and affected nerve-related variables

Potential determinants	Pain (None, mild, moderate, severe, extreme)	
	ŷ	*p*-values
Amputation and prosthesis		
Aetiology (traumatic)	0.851	< 0.000*
Time since amputation	0.580	< 0.000*
Prosthetic wear time (days/week)	–0.684	0.033*
Prosthetic wear time (hours/day)	–0.272	0.244
Walking aid (walker)	–0.688	0.003*
Functional ability		
K-level	0.431	0.166
PDI	0.786	< 0.000*
LCI-5	0.325	0.151
Neuromas		
Number (0–3) (*n* = 22)	0.305	0.163
Affected nerve		
Common fibular (*n* = 4)	0.941	0.006*
Superficial fibular (*n* = 11)	–0.350	0.289
Deep fibular (*n* = 6)	–0.108	0.749
Tibial (*n* = 5)	0.322	0.421
Sural (*n* = 1)	1.000	0.284

* *p* ≤ 0.05. LCI-5: Locomotor Capabilities Index – 5; PDI: Pain Disability Index.

Lastly, the sonopalpation Tinel sign elicited a positive response for 10 of the 27 neuromas (37%). Only a positive response on the superficial fibular nerve was statistically significantly associated with having moderate to extreme pain (X2(1) = 7.219, *p* = 0.007). No association was found for the deep fibular nerve (X^2^(1) = 0.375, *p* = 0.540), or the tibial nerve (X^2^(1) = 2.222, *p* = 0.136). Furthermore, all participants with a neuroma on the common fibular nerve or the sural nerve expressed moderate-to-extreme pain, which again prohibited group comparison and hypothesis testing.

## DISCUSSION

The results of the present study support the hypotheses in part. It was hypothesized that higher pain levels would be strongly or very strongly associated with less prosthetic use, lower functional capacity, and less participation in activities. The current results confirmed that the greater the pain intensity at the residual limb the less prone the individuals were to wear their prosthesis on a weekly basis and the more participation in essential life activities was reduced. Notably, pain intensity in the residual limb only minimally affected participation in essential life activities. This is evidenced by the fact that participants with a moderate-to-extreme pain score reported a median score of only 12 (out of 70) on the PDI.

Conversely, the current results did not reveal a relationship between pain intensity in the residual limb and functional capacity, particularly locomotor activities measured with the K-levels and LCI-5 scores. Indeed, participants in both groups had high LCI-5 scores, even those experiencing the higher levels on the NPRS. While experiencing pain and taking sitting breaks when needed, participants were still able to achieve higher functional tasks, such as walking on uneven terrain, and did not require the use of walking aids. Amputation-related pain of different aetiologies, i.e., phantom limb pain, RLP, and other regional pain, such as back pain, has been extensively described and its frequency has been reported within the literature (22, 23). However, very little is known regarding its effect on functional performance, especially for RLP. Ehde et al. (22) investigated phantom limb pain and RLP among other sources of pain in a sample of participants with lower limb amputations. They reported that 33% of their participants described their RLP as “extremely bothersome”. While the authors quantified the disability associated with phantom limb pain (23% of their sample reported high disability and moderate to severe limitation from their phantom limb pain), this outcome was not evaluated for RLP. Furthermore, although a small number of studies have examined mobility predictors in individuals with lower limb amputation, such as age and medical comorbidities, RLP has not previously been examined (24, 25). Although the literature has demonstrated that RLP was an important factor affecting quality of life (26), its impact on functional ability must be explored further, to ensure that an understanding of how RLP impacts functional ability is better integrated into assessments, treatments, and rehabilitation plans.

The results of the present study do not entirely support the secondary hypothesis. In fact, ultrasound biomarkers did not differ between the 2 pain intensity subgroups. Indeed, the number of neuromas, the CSA of the neuroma, the thickness ratio of the neuroma, and the depth of soft tissue covering the neuroma were all found to have no statistically significant impact on the highest level of pain experienced in the last week. These results align with previous literature in respect of the number (4) and size of neuromas (4, 27), where no significant association between these parameters and pain was found. Such findings may initially be surprising, given that there is a common belief among clinicians that features such as size or total count can have an impact on how painful something is. However, only limited and relatively weak research evidence is available to support such beliefs in the context of neuromas. Furthermore, a second clinical belief is that the greater the thickness of the soft tissue covering a neuroma, the less chance a person may have to be affected by pain. Such a potential protective effect was not supported by the results of the present study. However, the current study demonstrated that a neuroma on the common fibular nerve is very strongly associated with a higher pain score. It is possible that the proximity of the common fibular nerve to the fibular head may be a source of conflict and compression, and therefore pain. These results should be interpreted with caution given the small number of participants.

The results of the present study also do support the hypothesis that higher pain level was expected to be strongly associated with the presence of symptoms upon neuroma compression. Participants with moderate-to-extreme pain intensity were symptomatic (i.e., a positive sonopalpation Tinel sign) upon superficial fibular nerve neuroma compression, the nerve with the greatest number of neuromas in our study. An easily irritated neuroma, as by a slight external pressure reproduced with the transducer, may lead to daily pain in the socket or on any other surface. Although a small number of studies have used a similar sonographic Tinel sign to identify symptomatic neuromas (27) or other peripheral nerve abnormalities (28–30), the present study is the first to link the sonopalpation Tinel sign with everyday pain.

### Study limitations

In the context of this exploratory associational study, the small sample size confirms the relevance of the main constructs investigated but uncertainties exist surrounding the best determinants, and the best predictors of pain have yet to be confirmed using an ordinal logistic regression and variance inflation ratio to measure the degree of collinearity across selected variables. In order to do so and strengthen level of evidence, a large observational epidemiologic study with a minimum total sample size of 80 people who sustained a transtibial amputation and report (*experimental*) or do not report (*control*) neuropathic RLP is needed. Refining the neuroma-related determinants using biomarkers obtained via quantitative musculoskeletal ultrasound imaging should also be considered in a large observational epidemiologic study. Some technical challenges while conducting the imaging examination of these neuromas, due in part to the underuse, atrophy, or fatty metaplasia of the remaining calf muscle, also deserve attention. This is especially true for the distal part of the tibial nerve. Furthermore, post-amputation complications can impair multiple body functions and structures, leading to activity limitations and participation restrictions. Residual limb pain can arise from different causes beyond neuromas, which typically results in neuropathic pain when symptomatic. Differentiating between neuropathic and somatic pain can sometimes be complex. The use of a specific diagnostic instrument, such as the DN4 questionnaire, with a scoring system for diagnosing neuropathic pain could improve diagnostic accuracy and is encouraged in future studies (31). Additionally, a psychological index as an outcome measure might have been a pertinent addition to the comprehensive biopsychosocial model of pain and therefore have offered a better understanding of the experience of individuals living with amputations. Lastly, caution is recommended when inferring from the present results as no valid assumptions concerning causative factors can be made given the study design and the strength of the evidence generated.

### Conclusion

The results of the present exploratory study support the hypothesis that an association exists between the absence of a walking aid, less daily prosthesis wearing time, a higher PDI score, and the presence of pain upon compression of superficial fibular nerve neuroma with moderate-to-extreme neuropathic RLP. Features of the neuroma characterized via ultrasound imaging biomarkers, including soft tissue covering the neuroma, were not associated with neuropathic RLP intensity. Careful interpretation of the results is recommended considering the strength of the evidence and remaining uncertainties and additional research is essential to strengthen the evidence.
